# Supplementary material for: Geographic origin and timing of colonization of the Pacific Coast of North America by the rocky shore gastropod Littorina sitkana
Source: PeerJ. 2019 Nov 4;7:e7987. doi: 10.7717/peerj.7987 (PMC6836758; doi:10.7717/peerj.7987)
Supplement: Table S1 — ABC model uniform prior distributions for demographic parameters. [file peerj-07-7987-s001.docx]

**Table S1** **ABC prior distributions.** ABC model uniform prior distributions for demographic parameters. Also see Figure 1.

**Model parameter Minimum Maximum**

COR *N_e_* 10 200,000

KOD *N_e_* 10 200,000

JUN *N_e_* 10 200,000

PET *N_e_* 10 500,000

ERI *N_e_* 10 500,000

KHO *N_e_* 10 500,000

STA *N_e_* 10 500,000

*t_a_* 10 50,000

*t_b_* 10 200,000

*t_c_* 10 300,000

*t_d_* 10 400,000

*t_e_* 10 500,000

*t_f_* 10 600,000
