# Supplementary material for: Geographic origin and timing of colonization of the Pacific Coast of North America by the rocky shore gastropod Littorina sitkana
Source: PeerJ. 2019 Nov 4;7:e7987. doi: 10.7717/peerj.7987 (PMC6836758; doi:10.7717/peerj.7987)
Supplement: Table S2 — Mitochondrial cytochrome b (CytB) sample sizes (N, number of haplotypes sequenced), haplotype diversity (h), nucleotide diversity (π), Tajima’s D, Fu’s Fs, and Ramos-Onsins and Rozas’ R2. Standard deviations in parentheses. Boldface indicates statistically significant values. See Table 1 for sample locality abbreviations. NWP: Northwest Pacific; NEP: Northeast Pacific. [file peerj-07-7987-s002.docx]

**Table S2** **MtDNA sequence diversity statistics.** Mitochondrial cytochrome b (*CYTB*) sample sizes (*N*, number of haplotypes sequenced), haplotype diversity (*h*), average number of pairwise differences (*k*), nucleotide diversity (*π*), Tajima’s *D*, Fu’s *F_s_*, and Ramos-Onsins and Rozas’ *R_2_*. Standard deviations in parentheses. Boldface indicates statistically significant values. See Table 1 for sample locality abbreviations. NWP: Northwest Pacific; NEP: Northeast Pacific.

| **Locality** | **Region** | ***N*** | ***h*** | ***π*** | ***D*** | ***F_s_*** | ***R_2_*** |
| --- | --- | --- | --- | --- | --- | --- | --- |
| VOS | NWP | 17 | 0.757 (0.071) | 0.004 (0.003) | 0.271 | -0.151 | 0.156 |
| ERI | NWP | 11 | 0.345 (0.172) | 0.002 (0.002) | **-1.791** | 0.401 | 0.231 |
| NEM | NWP | 14 | 0.275 (0.148) | 0.0007 (0.0008) | -1.481 | **-1.475** | 0.175 |
| UTO | NWP | 9 | 0.639 (0.126) | 0.006 (0.004) | 1.265 | 2.224 | 0.226 |
| KHO | NWP | 16 | 0.675 (0.062) | 0.003(0.002) | 0.788 | 1.313 | 0.192 |
| STA | NWP | 8 | 0.000 | 0.000 | NA | NA | NA |
| PET | NWP | 16 | 0.325 (0.125) | 0.003 (0.002) | 0.245 | 3.430 | 0.163 |
| KOD | NEP | 9 | 0.000 | 0.000 | NA | NA | NA |
| COR | NEP | 12 | 0.439 (0.158) | 0.001 (0.001) | -0.85 | -0.725 | 0.158 |
| JUN | NEP | 8 | 0.000 | 0.000 | NA | NA | NA |
| RUP | NEP | 14 | 0.143 (0.119) | 0.0003 (0.0006) | -1.155 | -0.595 | 0.100 |
| CAM | NEP | 11 | 0.000 | 0.000 | NA | NA | NA |
| REN | NEP | 6 | 0.000 | 0.000 | NA | NA | NA |
| SJI | NEP | 6 | 0.000 | 0.000 | NA | NA | NA |
