# Supplementary material for: Geographic origin and timing of colonization of the Pacific Coast of North America by the rocky shore gastropod Littorina sitkana
Source: PeerJ. 2019 Nov 4;7:e7987. doi: 10.7717/peerj.7987 (PMC6836758; doi:10.7717/peerj.7987)
Supplement: Table S3 — Nuclear-encoded adenosine triphosphate synthetase subunit a intron sample sizes (N, number of haplotypes sequenced), haplotype diversity (h), nucleotide diversity (π), Tajima’s D, Fu’s Fsstatistic, and Ramos-Onsins and Rozas’ R2 statistic. Standard deviations in parentheses. Boldface indicates statistically significant values. See Table 1 for sample locality abbreviations. NWP: Northwest Pacific; NEP: Northeast Pacific. [file peerj-07-7987-s003.docx]

**Table S3** **Nuclear *ATPSα* sequence diversity statistics.** Nuclear-encoded adenosine triphosphate synthetase subunit α (*ATPSα*) intron sample sizes (*N*, number of haplotypes sequenced), haplotype diversity (*h*), average number of pairwise differences (*k*), nucleotide diversity (*π*), Tajima’s D, Fu’s *F_s_* statistic, and Ramos-Onsins and Rozas’ *R_2_* statistic. Standard deviations in parentheses. Boldface indicates statistically significant values. See Table 1 for sample locality abbreviations. NWP: Northwest Pacific; NEP: Northeast Pacific.

| **Locality** | **Region** | ***N*** | ***h*** | ***π*** | ***D*** | ***F_s_*** | ***R_2_*** |
| --- | --- | --- | --- | --- | --- | --- | --- |
| ERI | NWP | 8 | 0.750 (0.096) | 0.008 (0.004) | 0.756 | 1.837 | 0.225 |
| KHO | NWP | 7 | 0.286 (0.196) | 0.002 (0.002) | -1.237 | 0.856 | 0.350 |
| STA | NWP | 7 | 0.484 (0.142) | 0.004 (0.002) | -1.314 | 0.969 | 0.132 |
| PET | NWP | 8 | 0.464 (0.200) | 0.003 (0.002) | -1.448 | -0.305 | 0.232 |
| KOD | NEP | 9 | 0.000 | 0.000 | NA | NA | NA |
| COR | NEP | 8 | 0.250 (0.180) | 0.008 (0.003) | **-1.723** | 3.887 | 0.331 |
| JUN | NEP | 8 | 0.536 (0.123) | 0.002 (0.002) | 1.167 | 0.866 | 0.268 |
