# Supplementary material for: Geographic origin and timing of colonization of the Pacific Coast of North America by the rocky shore gastropod Littorina sitkana
Source: PeerJ. 2019 Nov 4;7:e7987. doi: 10.7717/peerj.7987 (PMC6836758; doi:10.7717/peerj.7987)
Supplement: Table S4 — Nuclear-encoded adenosine triphosphate synthetase subunit b intron sample sizes (N, number of haplotypes sequenced), haplotype diversity (h), nucleotide diversity (π), Tajima’s D, Fu’s Fs statistic, and Ramos-Onsins and Rozas’ R2 statistic. Standard deviations in parentheses. Boldface indicates statistically significant values. See Table 1 for sample locality abbreviations. NWP: Northwest Pacific; NEP: Northeast Pacific. [file peerj-07-7987-s004.docx]

**Table S4** **Nuclear *ATPSβ* sequence diversity statistics.** Nuclear-encoded adenosine triphosphate synthetase subunit β (*ATPSβ*) intron sample sizes (*N*, number of haplotypes sequenced), haplotype diversity (*h*), average number of pairwise differences (*k*), nucleotide diversity (*π*), Tajima’s *D*, Fu’s *F_s_* statistic, and Ramos-Onsins and Rozas’ *R_2_* statistic. Standard deviations in parentheses. Boldface indicates statistically significant values. See Table 1 for sample locality abbreviations. NWP: Northwest Pacific; NEP: Northeast Pacific.

| Locality | Region | *N* | *h* | *π* | *D* | *F_s_* | *R_2_* |
| --- | --- | --- | --- | --- | --- | --- | --- |
| ERI | NWP | 10 | 0 | 0 | NA | NA | NA |
| KHO | NWP | 9 | 0.533 (0.172) | 0.0021 (0.002) | 1.031 | 1.723 | 0.267 |
| STA | NWP | 7 | 0.429 (0.169) | 0.0014 (0.002) | 0.414 | 1.653 | 0.214 |
| PET | NWP | 8 | 0.607 (0.164) | 0.0023 (0.001) | 0.585 | 0.723 | 0.209 |
| KOD | NEP | 8 | 0.250 (0.180) | 0.0004 (0.001) | -1.055 | -0.182 | 0.331 |
| COR | NEP | 8 | 0.250 (0.180) | 0.0004 (0.001) | -1.055 | -0.182 | 0.331 |
| JUN | NEP | 8 | 0.250 (0.180) | 0.0004 (0.001) | 0.001 | 0.233 | 0.205 |
