# Supplementary material for: Geographic origin and timing of colonization of the Pacific Coast of North America by the rocky shore gastropod Littorina sitkana
Source: PeerJ. 2019 Nov 4;7:e7987. doi: 10.7717/peerj.7987 (PMC6836758; doi:10.7717/peerj.7987)
Supplement: Table S6 — Boldface values indicate NEP versus NWP population comparisons. * P < 0.05. [file peerj-07-7987-s006.docx]

**Table S6 Pairwise estimates of *Φ_ST_* for mitochondrial *CYTB***. Boldface values indicate NEP versus NWP population comparisons.

|  | VOS | ERI | NEM | UTO | KHO | STA | PET | KOD | COR | JUN | RUP | CAM | REN | SJI |
| --- | --- | --- | --- | --- | --- | --- | --- | --- | --- | --- | --- | --- | --- | --- |
| VOS | - |  |  |  |  |  |  |  |  |  |  |  |  |  |
| ERI | 0.541* | - |  |  |  |  |  |  |  |  |  |  |  |  |
| NEM | 0.652* | 0.011 | - |  |  |  |  |  |  |  |  |  |  |  |
| UTO | 0.284* | 0.259* | 0.404* | - |  |  |  |  |  |  |  |  |  |  |
| KHO | 0.253* | 0.534* | 0.655* | 0.259* | - |  |  |  |  |  |  |  |  |  |
| STA | 0.223* | 0.752* | 0.915* | 0.414* | 0.564* | - |  |  |  |  |  |  |  |  |
| PET | 0.210* | 0.470* | 0.619* | 0.272* | 0.408* | 0.061 | - |  |  |  |  |  |  |  |
| KOD | **0.237*** | **0.763*** | **0.919*** | **0.434*** | **0.576*** | **0.000** | **0.073** | - |  |  |  |  |  |  |
| COR | **0.243*** | **0.699*** | **0.839*** | **0.417*** | **0.547*** | **0.017** | **0.087** | 0.030 | - |  |  |  |  |  |
| JUN | **0.223*** | **0.752*** | **0.915*** | **0.414*** | **0.564*** | **0.000** | **0.061** | 0.000 | 0.017 | - |  |  |  |  |
| RUP | **0.278*** | **0.776*** | **0.903*** | **0.491*** | **0.605*** | **-0.045** | **0.110** | -0.035 | 0.055 | -0.045 | - |  |  |  |
| CAM | **0.261*** | **0.783*** | **0.925*** | **0.470*** | **0.600*** | **0.000** | **0.094** | 0.000 | 0.051 | 0.000 | -0.018 | - |  |  |
| REN | **0.189*** | **0.725*** | **0.906*** | **0.365*** | **0.534*** | **0.000** | **0.029** | 0.000 | -0.018 | 0.000 | -0.075 | 0.000 | - |  |
| SJI | **0.189*** | **0.725*** | **0.906*** | **0.365*** | **0.534*** | **0.000** | **0.029** | 0.000 | -0.018 | 0.000 | -0.075 | 0.000 | 0.000 | - |

* *P* < 0.05.
