# Supplementary material for: Geographic origin and timing of colonization of the Pacific Coast of North America by the rocky shore gastropod Littorina sitkana
Source: PeerJ. 2019 Nov 4;7:e7987. doi: 10.7717/peerj.7987 (PMC6836758; doi:10.7717/peerj.7987)
Supplement: Supplemental Information 7 — Boldface values indicate NEP versus NWP population comparisons. * P < 0.05. [file peerj-07-7987-s007.docx]

**Table S7** **Pairwise estimates of *Φ_ST_* for nuclear-encoded *ATPSα*.** Boldface values indicate NEP versus NWP population comparisons.

|  | ERI | KHO | STA | PET | KOD | COR | JUN |
| --- | --- | --- | --- | --- | --- | --- | --- |
| ERI | - |  |  |  |  |  |  |
| KHO | 0.195 | - |  |  |  |  |  |
| STA | 0.065* | -0.005 | - |  |  |  |  |
| PET | -0.003 | 0.822* | 0.707* | - |  |  |  |
| KOD | **-1.026** | **0.918*** | **0.768*** | **-0.079** | - |  |  |
| COR | **0.007** | **0.647*** | **0.598*** | **0.253*** | 0.134* | - |  |
| JUN | **-0.002** | **0.856*** | **0.725*** | **0.121** | 0.318 | 0.321 | - |

* *P* < 0.05.
