# Supplementary material for: Geographic origin and timing of colonization of the Pacific Coast of North America by the rocky shore gastropod Littorina sitkana
Source: PeerJ. 2019 Nov 4;7:e7987. doi: 10.7717/peerj.7987 (PMC6836758; doi:10.7717/peerj.7987)
Supplement: Table S8 — Boldface values indicate NEP versus NWP population comparisons * P < 0.05. [file peerj-07-7987-s008.docx]

**Table S8** **Pairwise estimates of *Φ_ST_* for nuclear-encoded *ATPSβ*.** Boldface values indicate NEP versus NWP population comparisons

|  | ERI | KHO | STA | PET | KOD | COR | JUN |
| --- | --- | --- | --- | --- | --- | --- | --- |
| ERI | - |  |  |  |  |  |  |
| KHO | 0.398* | - |  |  |  |  |  |
| STA | 0.119* | 0.232* | - |  |  |  |  |
| PET | 0.403* | 0.363* | 0.028 | - |  |  |  |
| KOD | **0.448*** | **0.372*** | **0.033** | **0.171** | - |  |  |
| COR | **0.434*** | **0.372*** | **0.033** | **0.171** | 0.0045 | - |  |
| JUN | **0.448*** | **0.372*** | **0.033** | **0.171** | 0.0045 | 0.0067 | - |

* *P* < 0.05.
