# Supplementary material for: Geographic origin and timing of colonization of the Pacific Coast of North America by the rocky shore gastropod Littorina sitkana
Source: PeerJ. 2019 Nov 4;7:e7987. doi: 10.7717/peerj.7987 (PMC6836758; doi:10.7717/peerj.7987)
Supplement: Table S9 — Boldface values indicate NEP versus NWP population comparisons. [file peerj-07-7987-s009.docx]

**Table S9** **Pairwise estimates of *Φ_ST_* for nuclear-encoded *APN54*.** Boldface values indicate NEP versus NWP population comparisons.

|  | ERI | KHO | STA | PET | KOD | COR | JUN |
| --- | --- | --- | --- | --- | --- | --- | --- |
| ERI | - |  |  |  |  |  |  |
| KHO | 0.200 | - |  |  |  |  |  |
| STA | 0.094 | -0.151 | - |  |  |  |  |
| PET | 0.470* | 0.409* | 0.419* | - |  |  |  |
| KOD | **0.377** | **0.308*** | **0.265*** | **0.501*** | - |  |  |
| COR | **0.094** | **0.209** | **0.143** | **0.461*** | -0.061 | - |  |
| JUN | **0.010** | **0.261** | **0.143** | **0.519*** | 0.347 | 0.020 | - |

* *P* < 0.05.
