# Supplementary material for: Geographic origin and timing of colonization of the Pacific Coast of North America by the rocky shore gastropod Littorina sitkana
Source: PeerJ. 2019 Nov 4;7:e7987. doi: 10.7717/peerj.7987 (PMC6836758; doi:10.7717/peerj.7987)
Supplement: Table S10 — Best 100 tree topologies ranked by the product of the posterior clade probabilities (PPCP) for diverged populations of Littorina sitkana. The sampled populations in each Newick-formatted tree topology are numbered 0 to 6 (COR, KOD, JUN, PET, ERI, STA, KHO, respectively) with ancestral populations numbered 7 to 12, ordered numerically as they coalesce going backwards in time. Note that each of the top 100 trees have a clade consisting of the three northeastern Pacific populations (0-COR, 1-KOD, 2-JUN) and the easternmost population in the northwestern Pacific (3-PET), indicated by boldface. [file peerj-07-7987-s010.docx]

**Table S10**. **IMa3 tree topology selection.** Best 100 tree topologies ranked by the product of the posterior clade probabilities PPCP) for diverged populations of *Littorina sitkana*. The sampled populations in each Newick-formatted tree topology are numbered 0 to 6 (COR, KOD, JUN, PET, ERI, STA, KHO, respectively) with ancestral populations numbered 7 to 12, ordered numerically as they coalesce going backwards in time. Note that each of the top 100 trees have a clade consisting of the three northeastern Pacific populations (0-COR, 1-KOD, 2-JUN) and the easternmost population in the northwestern Pacific (3-PET), indicated by boldface.

| Tree Topology | PPCP |
| --- | --- |

| 1 | (6,(**(3,(2,(0,1)7)8)**9,(4,5)10)11)12 | 0.0086767 |
| --- | --- | --- |
| 2 | (6,((4,5)9,**(3,(2,(0,1)7)8)**10)11)12 | 0.0086767 |
| 3 | (6,((4,5)8,**(3,(2,(0,1)7)9)**10)11)12 | 0.0086767 |
| 4 | (6,((4,5)7,**(3,(2,(0,1)8)**9)10)11)12 | 0.0086767 |
| 5 | ((6,**(3,(2,(0,1)7)8)**9)10,(4,5)11)12 | 0.0078449 |
| 6 | ((4,5)10,(6,**(3,(2,(0,1)7)8)**9)11)12 | 0.0078449 |
| 7 | ((4,5)9,(6,**(3,(2,(0,1)7)8)**10)11)12 | 0.0078449 |
| 8 | ((4,5)8,(6,**(3,(2,(0,1)**7)9)10)11)12 | 0.0078449 |
| 9 | ((4,5)7,(6,**(3,(2,(0,1)**8)9)10)11)12 | 0.0078449 |
| 10 | (**(3,(2,(0,1)7)8)**9,(6,(4,5)10)11)12 | 0.0069545 |
| 11 | (**(3,(2,(0,1)7)8)**10,(6,(4,5)9)11)12 | 0.0069545 |
| 12 | (**(3,(2,(0,1)7)9)**10,(6,(4,5)8)11)12 | 0.0069545 |
| 13 | (**(3,(2,(0,1)8)9)**10,(6,(4,5)7)11)12 | 0.0069545 |
| 14 | ((6,(4,5)9)10,**(3,(2,(0,1)7)8)**11)12 | 0.0069545 |
| 15 | ((6,(4,5)8)10,**(3,(2,(0,1)7)9)**11)12 | 0.0069545 |
| 16 | ((6,(4,5)8)9,**(3,(2,(0,1)7)10)**11)12 | 0.0069545 |
| 17 | ((6,(4,5)7)10,**(3,(2,(0,1)8)9)**11)12 | 0.0069545 |
| 18 | ((6,(4,5)7)8,**(3,(2,(0,1)9)10)**11)12 | 0.0069545 |
| 19 | ((6,(4,5)7)9,**(3,(2,(0,1)8)10)**11)12 | 0.0069545 |
| 20 | (6,(**((0,1)7,(2,3)8)**9,(4,5)10)11)12 | 0.004986 |
| 21 | (6,(**((2,3)7,(0,1)8)**9,(4,5)10)11)12 | 0.004986 |
| 22 | (6,((4,5)9,**((0,1)7,(2,3)8)**10)11)12 | 0.004986 |
| 23 | (6,((4,5)8,**((0,1)7,(2,3)9)**10)11)12 | 0.004986 |
| 24 | (6,((4,5)9,**((2,3)7,(0,1)8)**10)11)12 | 0.004986 |
| 25 | (6,((4,5)7,**((0,1)8,(2,3)9)**10)11)12 | 0.004986 |
| 26 | (6,((4,5)8,**((2,3)7,(0,1)9)**10)11)12 | 0.004986 |
| 27 | (6,((4,5)7,**((2,3)8,(0,1)9)**10)11)12 | 0.004986 |
| 28 | ((4,5)10,(6,**((0,1)7,(2,3)8)**9)11)12 | 0.004508 |
| 29 | ((4,5)9,(6,**((0,1)7,(2,3)8)**10)11)12 | 0.004508 |
| 30 | ((4,5)10,(6,**((2,3)7,(0,1)8)**9)11)12 | 0.004508 |
| 31 | ((4,5)8,(6,**((0,1)7,(2,3)9)**10)11)12 | 0.004508 |
| 32 | ((4,5)9,(6,**((2,3)7,(0,1)8)**10)11)12 | 0.004508 |
| 33 | ((4,5)7,(6,**((0,1)8,(2,3)9)**10)11)12 | 0.004508 |
| 34 | ((4,5)8,(6,**((2,3)7,(0,1)9)**10)11)12 | 0.004508 |
| 35 | ((4,5)7,(6,**((2,3)8,(0,1)9)**10)11)12 | 0.004508 |
| 36 | ((6,**((0,1)7,(2,3)8)**9)10,(4,5)11)12 | 0.004508 |
| 37 | ((6,**((2,3)7,(0,1)8)**9)10,(4,5)11)12 | 0.004508 |
| 38 | (6,(**(3,(0,(1,2)7)8)**9,(4,5)10)11)12 | 0.0042494 |
| 39 | (6,((4,5)9,**(3,(0,(1,2)7)8)**10)11)12 | 0.0042494 |
| 40 | (6,((4,5)8,**(3,(0,(1,2)7)9)**10)11)12 | 0.0042494 |
| 41 | (6,((4,5)7,**(3,(0,(1,2)8)9)**10)11)12 | 0.0042494 |
| 42 | ((6,(4,5)9)10,**((0,1)7,(2,3)8)**11)12 | 0.0039963 |
| 43 | ((6,(4,5)8)10,**((0,1)7,(2,3)9)**11)12 | 0.0039963 |
| 44 | ((6,(4,5)8)9,**((0,1)7,(2,3)10)**11)12 | 0.0039963 |
| 45 | ((6,(4,5)7)10,**((0,1)8,(2,3)9)**11)12 | 0.0039963 |
| 46 | ((6,(4,5)7)9,**((0,1)8,(2,3)10)**11)12 | 0.0039963 |
| 47 | ((6,(4,5)7)8,**((0,1)9,(2,3)10)**11)12 | 0.0039963 |
| 48 | (**((0,1)7,(2,3)8)**9,(6,(4,5)10)11)12 | 0.0039963 |
| 49 | (**((0,1)7,(2,3)8)**10,(6,(4,5)9)11)12 | 0.0039963 |
| 50 | (**((0,1)7,(2,3)9)**10,(6,(4,5)8)11)12 | 0.0039963 |
| 51 | (**((2,3)7,(0,1)8)**9,(6,(4,5)10)11)12 | 0.0039963 |
| 52 | (**((2,3)7,(0,1)8)**10,(6,(4,5)9)11)12 | 0.0039963 |
| 53 | ((6,(4,5)9)10,**((2,3)7,(0,1)8)**11)12 | 0.0039963 |
| 54 | (**((0,1)8,(2,3)9)**10,(6,(4,5)7)11)12 | 0.0039963 |
| 55 | (**((2,3)7,(0,1)9)**10,(6,(4,5)8)11)12 | 0.0039963 |
| 56 | ((6,(4,5)8)10,**((2,3)7,(0,1)9)**11)12 | 0.0039963 |
| 57 | (**((2,3)8,(0,1)9)**10,(6,(4,5)7)11)12 | 0.0039963 |
| 58 | ((6,(4,5)8)9,**((2,3)7,(0,1)10)**11)12 | 0.0039963 |
| 59 | ((6,(4,5)7)10,**((2,3)8,(0,1)9)**11)12 | 0.0039963 |
| 60 | ((6,(4,5)7)9,**((2,3)8,(0,1)10)**11)12 | 0.0039963 |
| 61 | ((6,(4,5)7)8,**((2,3)9,(0,1)10)**11)12 | 0.0039963 |
| 62 | ((6,**(3,(0,(1,2)7)8)**9)10,(4,5)11)12 | 0.003842 |
| 63 | ((4,5)10,(6,**(3,(0,(1,2)7)8)**9)11)12 | 0.003842 |
| 64 | ((4,5)9,(6,**(3,(0,(1,2)7)8)**10)11)12 | 0.003842 |
| 65 | ((4,5)8,(6,**(3,(0,(1,2)7)9)**10)11)12 | 0.003842 |
| 66 | ((4,5)7,(6,**(3,(0,(1,2)8)9)**10)11)12 | 0.003842 |
| 67 | (6,(**(3,(1,(0,2)7)8)**9,(4,5)10)11)12 | 0.0035206 |
| 68 | (6,((4,5)9,**(3,(1,(0,2)7)8)**10)11)12 | 0.0035206 |
| 69 | (6,((4,5)8,**(3,(1,(0,2)7)9)**10)11)12 | 0.0035206 |
| 70 | (6,((4,5)7,**(3,(1,(0,2)8)9)**10)11)12 | 0.0035206 |
| 71 | (**(3,(0,(1,2)7)8)**9,(6,(4,5)10)11)12 | 0.0034059 |
| 72 | (**(3,(0,(1,2)7)8)**10,(6,(4,5)9)11)12 | 0.0034059 |
| 73 | ((6,(4,5)9)10,**(3,(0,(1,2)7)8)**11)12 | 0.0034059 |
| 74 | (**(3,(0,(1,2)8)9)**10,(6,(4,5)7)11)12 | 0.0034059 |
| 75 | (**(3,(0,(1,2)7)9)**10,(6,(4,5)8)11)12 | 0.0034059 |
| 76 | ((6,(4,5)7)10,**(3,(0,(1,2)8)9)**11)12 | 0.0034059 |
| 77 | ((6,(4,5)8)9,**(3,(0,(1,2)7)10)**11)12 | 0.0034059 |
| 78 | ((6,(4,5)8)10,**(3,(0,(1,2)7)9)**11)12 | 0.0034059 |
| 79 | ((6,(4,5)7)9,**(3,(0,(1,2)8)10)**11)12 | 0.0034059 |
| 80 | ((6,(4,5)7)8,**(3,(0,(1,2)9)10)**11)12 | 0.0034059 |
| 81 | ((4,5)10,(6,**(3,(1,(0,2)7)8)**9)11)12 | 0.003183 |
| 82 | ((4,5)9,(6,**(3,(1,(0,2)7)8)**10)11)12 | 0.003183 |
| 83 | ((6,**(3,(1,(0,2)7)8)**9)10,(4,5)11)12 | 0.003183 |
| 84 | ((4,5)8,(6,**(3,(1,(0,2)7)9)**10)11)12 | 0.003183 |
| 85 | ((4,5)7,(6,**(3,(1,(0,2)8)9)**10)11)12 | 0.003183 |
| 86 | (**(3,(1,(0,2)7)8)**9,(6,(4,5)10)11)12 | 0.0028218 |
| 87 | (**(3,(1,(0,2)7)8)**10,(6,(4,5)9)11)12 | 0.0028218 |
| 88 | ((6,(4,5)9)10,**(3,(1,(0,2)7)8)**11)12 | 0.0028218 |
| 89 | (**(3,(1,(0,2)7)9)**10,(6,(4,5)8)11)12 | 0.0028218 |
| 90 | (**(3,(1,(0,2)8)9)**10,(6,(4,5)7)11)12 | 0.0028218 |
| 91 | ((6,(4,5)8)10,**(3,(1,(0,2)7)9)**11)12 | 0.0028218 |
| 92 | ((6,(4,5)7)10,**(3,(1,(0,2)8)9)**11)12 | 0.0028218 |
| 93 | ((6,(4,5)8)9,**(3,(1,(0,2)7)10)**11)12 | 0.0028218 |
| 94 | ((6,(4,5)7)9,**(3,(1,(0,2)8)10)**11)12 | 0.0028218 |
| 95 | ((6,(4,5)7)8,**(3,(1,(0,2)9)10)**11)12 | 0.0028218 |
| 96 | (**(3,(2,(0,1)7)8)**9,(5,(4,6)10)11)12 | 0.0023733 |
| 97 | (**(3,(2,(0,1)7)8)**10,(5,(4,6)9)11)12 | 0.0023733 |
| 98 | (**(3,(2,(0,1)7)9)**10,(5,(4,6)8)11)12 | 0.0023733 |
| 99 | (**(3,(2,(0,1)8)9)**10,(5,(4,6)7)11)12 | 0.0023733 |
| 100 | ((5,(4,6)9)10,**(3,(2,(0,1)7)8)**11)12 | 0.0023733 |
